# Wilson’s Disease with Lymphoproliferative Disorder: A Case Report

**DOI:** 10.31729/jnma.8983

**Published:** 2025-05-31

**Authors:** Ashish Jha, Saroj Kumar Shah, Ravi Ranjan Pradhan

**Affiliations:** 1 Department of Pediatrics, Madhesh Institute of Health Sciences, Janakpurdham, Dhanusha, Nepal; 2 Department of Pediatrics, Kanti Childrens' Hospital, Maharajgunj, Kathmandu, Nepal; 3 Beni Hospital, Beni, Myagdi, Nepal

**Keywords:** *hematological disorder*, *neurological manifestation*, *Wilson's disease*

## Abstract

Wilson's disease is characterized by copper accumulation in organs like liver, brain, and eyes, presenting with a varied clinical features, making it challenging to diagnose. This report describes a case of Wilson's disease with unusual hematological features. A 12-year-old from Himalayan region presented with weakness, dysarthria, tremors. Initial investigations revealed pancytopenia, and bone marrow showed a lymphoproliferative disorder. He developed behavioral changes, a flat affect, and scanning speech. Wilson's disease was confirmed through Kayser-Fleischer rings, reduced serum ceruloplasmin levels, and elevated urinary copper, supported by imaging.

## INTRODUCTION

Wilson's disease (WD) is an autosomal recessive disorder of copper metabolism with an estimated prevalence of 1 in 30,000 to 1 in 50,000 in the Asian population.^1^ Despite its well-defined diagnostic criteria, WD goes undiagnosed in the Southeast Asian regionlow and middle income countries due to limited access to advanced diagnostic options.^1^ The disease has various clinical manifestations including atypical presentations leading to delayed diagnosis and treatment, potentially resulting in severe complications even death.^2,3^ We describe a unique case of WD initially masquerading as primary hematological disorder which on subsequent evaluation and follow up revealed neuropsychiatric symptoms and uncommon hematological features. It reinstated the importance of considering atypical presentations of WD to ensure timely diagnosis and management.

## CASE PRESENTATION

A 12-year-old boy, developmentally normal, well-immunized according to the national immunization schedule, belonging to a poor socio-economic background residing in remote himalayan region of Nepal, born to non-consanguineous marriage, presented to the pediatric clinic with progressive limb weakness. The weakness initially made him unable to lift small objects and gradually worsened, leading to frequent falls while walking. Although there were no associated abnormal movements or episodes of fainting.

Two months later, the child developed alterations in speech that progressed to an inability to verbalize within a month accompanied by drooling of saliva. He also experienced difficulty grasping objects. His parents reported a decline in his scholastic performances. Further inquiry revealed the deterioration in performance was primarily due to progressive worsening of his handwriting, which became unrecognizable over the course of a month. Behavioral changes included increased stubbornness and incoherence during this period.

He had a significant family history since his older sibling died at 12 years of age due to an undiagnosed illness, reportedly characterized by abdominal swelling and jaundice. His younger sibling had been diagnosed with transfusion-dependent thalassemia, and both the parents were diagnosed with thalassemia minor.

On examination, the child was well-built, without dysmorphic features. Neurological evaluation revealed scanning speech and a normal gait, with no other cerebellar signs or focal neurological deficits. However, the child exhibited a flat affect and occasional agitating behavior, which had developed three months after the initial presentation. Systemic examination findings revealed splenomegaly. Initial laboratory investigations revealed pancytopenia, and a peripheral blood smear showed dimorphic anemia (normocytic and microcytic) (Table 1).

Bone marrow aspiration findings included lymphocytosis with dyserythropoiesis and increased megakaryopoiesis, raising the possibility of a lymphoproliferative disorder. Ultrasonography of the abdomen confirmed splenomegaly measuring 14.75 cm. Liver function tests were normal (Table 1).

**Table 1:** Laboratory investigations

Investigations:	Patient values	Reference values
Hemoglobin (g/dl)	10.1	Adult males: 13-16 g/dl, Adult Females: 12.1-15.1 g/dl, Children: 11.5-15.5 g/dl, New born: 13.5-24 g/dl
White blood count (cells/cumm)	1200	Adult males: 5000-10000/cumm, Adult females: 4500-11000/cumm, Children: 5000-10000/cumm, New born: 9000-30000/cumm
Platelet count (thousand/cumm)	40	Adult males: 135-317 thousand/cumm, Adult females: 157-371 thousand/cumm, Children: 250-450 thousand/cumm, New born: 150-450 thousand/cumm
Peripheral smear	Normocytic and microcytic red blood cells	Normocytic normochromic picture
Total Bilirubin (mg/dl)	1.1	Adults: 0.3-1.2mg/dl, Children: 0-1mg/dl
Direct Bilirubin (mg/dl)	0.1	<0.2 mg/dl
Albumin (g/dl)	3.9	3.5-5.2 g/dl
Aspartate aminotransferase (U/L)	22	Males: <50 U/L, Females: <35 U/L
Alanine aminotransferase (U/L)	28	Males: <50U/L, Females: <35U/L
Alkaline phosphatase (U/L)	49	Adults: 38-126 U/L, Children: 44-147 U/L
Direct Coomb's test	Negative	Negative
Serum ceruloplasmin (mg/dl)	18.8	20-60 mg/dl
24 hour urine copper (mcg)	57	3-50 mcg/day

cells/cumm: cells per cubic millimeter, mg/dl: milligram per deciliter, g/dl: gram per deciliter, U/L: Units per litermcg: microgram

Despite routine follow-ups, no clear explanation emerged for the neuropsychiatric manifestations until further neurological evaluations revealed the presence of a Kayser-Fleischer ring on slit-lamp examination. Additional tests showed a Direct Coomb's test negative, serum ceruloplasmin level of 18.8 mg/dl and, urinary copper of 57 mcg/24 hours (Table 1). Magnetic resonance imaging (MRI) of the brain demonstrated Fluid Attenuated Inversion Recovery (FLAIR) high-signal intensities in bilateral lentiform nucleus and thalamus, consistent with early manifestations of Wilson's disease (Figure 1).

The diagnosis of Wilson's disease was established based on the criteria from the 8th International Wilson's Disease Meeting, Leipzig, 2001.^2^ The child is currently on D-penicillamine at a dose 500 mg twice daily. The neuropsychiatric features have resolved and the child remains under regular follow-up.

## DISCUSSION

Wilson's disease is a rare autosomal recessive disorder of copper metabolism that leads to impaired copper excretion and its subsequent accumulation in various organs, most notably liver, brain, and eyes. The genetic basis of the disease involves pathogenic variants in the ATP7B gene located on chromosome 13, resulting in defective copper transport and excretion.^3^

The typical age of onset for WD is between 5 and 35 years, with hepatic manifestations predominating in the first decade and neurological manifestations more commonly presenting in the third decade of life.^4^ Our case aligns with this expected range, presenting with a combination of both hepatic and neuropsychiatric symptoms.

Notably, the child exhibited mood and behavioral changes, deteriorating handwriting, and declining academic performance which are common early neuropsychiatric manifestations in children with WD. The presence of drooling and speech abnormalities further supported the diagnosis, as these are characteristic features of the disease.^5^ Additionally, the child displayed a flat affect and incoherence in response to surrounding stimuli which are psychiatric manifestations that are less frequently encountered but still recognized in WD.^5^ Kayser Fleischer rings, a hallmark of WD, were present in this child and are consistent with almost all cases presenting with neuropsychiatric features.^6^ It is worth noting that the undiagnosed death of the child's elder sibling, who presented with ascites and jaundice, may have been attributed to delayed diagnosis of WD with hepatic involvement.

While hepatic and neuropsychiatric manifestations are the most common, hematological and renal involvement in WD is rare. Hemolytic anemia, typically Direct Coombs test-negative, is the most common hematological manifestation.^7^ However, our patient presented with pancytopenia, likely secondary to hypersplenism, which is an expected occurrence in WD due to portal hypertension. An interesting finding in our case was the co-incidence of a lymphoproliferative bone marrow disorder, a manifestation that is not commonly associated with WD. In addition, optical coherence tomography (OCT), a more precise technique for detecting copper deposition in the corneal stroma, could not be performed in our patient due to financial constraints. OCT is not only useful for diagnosis but also helps in monitoring treatment response over time.^6^

## CONCLUSION

Wilson's disease is a rare autosomal recessive disorder that presents with a diverse range of symptoms, often leading to misdiagnosis due to its multi-system involvement and different ages of presentations. Our case, with its unique hematological manifestation followed by the development of neurological symptoms, emphasizes the challenge of diagnosing Wilson's disease in resource-limited settings. While many features are typical, the unusual clinical signs in this case underscore the need for heightened awareness. In low- and middle-income countries like Nepal, financial constraints may limit the diagnostic options, making it crucial for clinicians to maintain a high index of suspicion. Early diagnosis is vital, as delayed recognition can lead to fatal outcomes, particularly when hepatic or neurological complications are involved.
